# Supplementary figures and images for: Establishment and Characterization of NCC-DDLPS4-C1: A Novel Patient-Derived Cell Line of Dedifferentiated Liposarcoma
Source: J Pers Med. 2021 Oct 24;11(11):1075. doi: 10.3390/jpm11111075 (PMC8618493; doi:10.3390/jpm11111075)

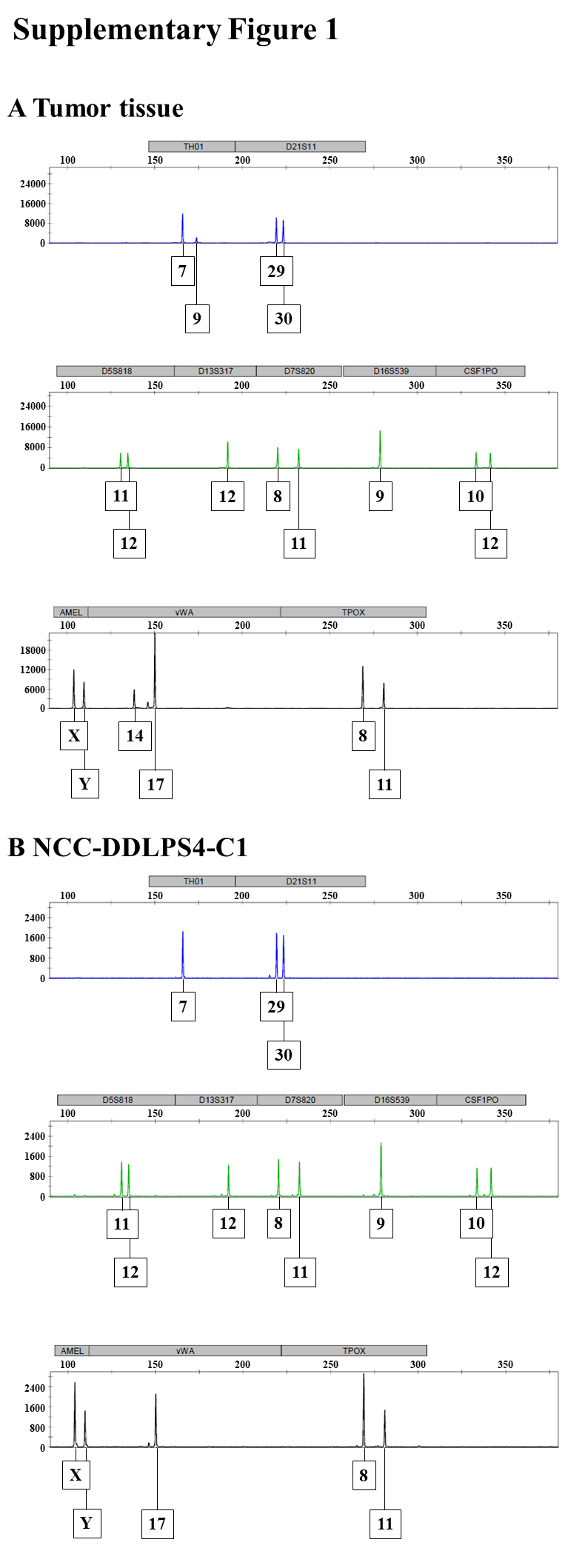

Supplement: Supplementary file 1 [file jpm-11-01075-s001.zip › Supplementary materials/Supplementary Figure 1 STR.tif]

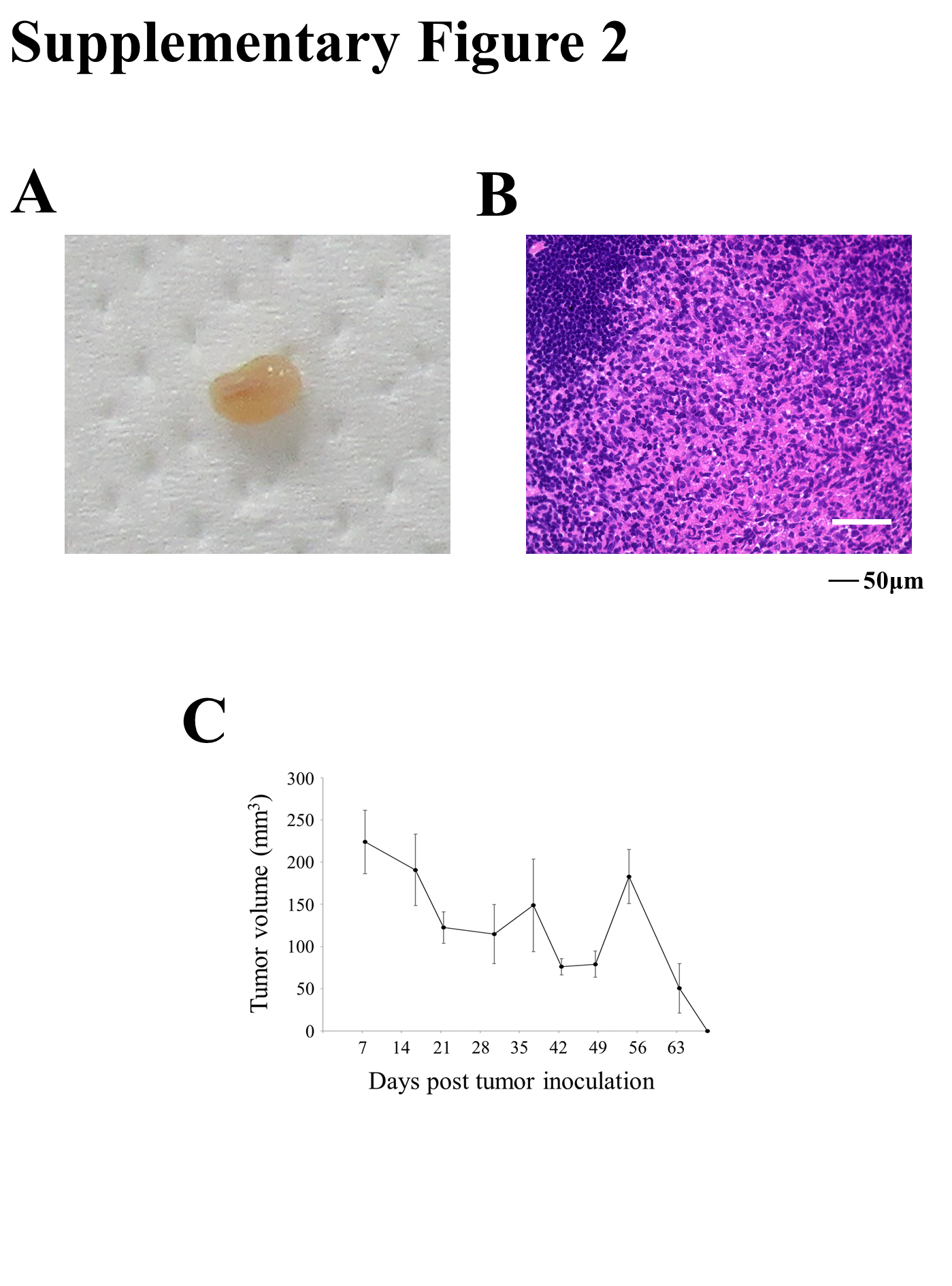

Supplement: Supplementary file 1 [file jpm-11-01075-s001.zip › Supplementary materials/Supplementary Figure 2 animal experiment.tif]

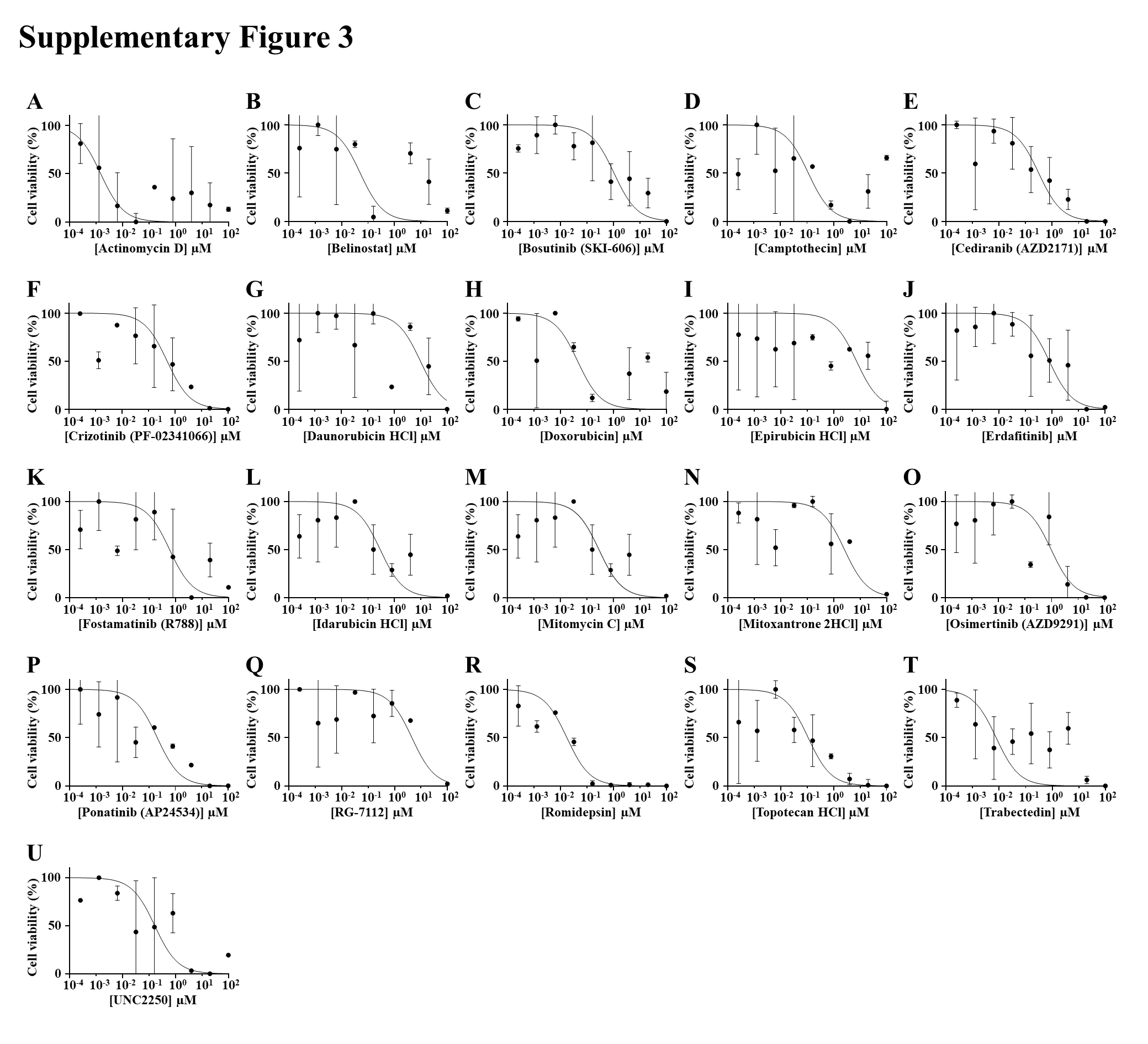

Supplement: Supplementary file 1 [file jpm-11-01075-s001.zip › Supplementary materials/Supplementary Figure 3 IC50 all.tif]
